# Knowledge, attitudes and practices of pregnant women for preventing risks and accidents with their newborns: cross-sectional study, Recife, 2024

**DOI:** 10.1590/S2237-96222026v35e20240822.en

**Published:** 2025-12-08

**Authors:** Maria Eduarda da Silva Bastos, Ana Júlia Falcão Nascimento, Maria Tereza Ferreira Soares, Maria Benita Alves da Silva Spinelli, Sandra Trindade Low

**Affiliations:** 1 Universidade de Pernambuco, Recife, PE, Brazil

**Keywords:** Health Knowledge, Attitudes and Practices, Accident Prevention, Infant, Newborn, Prenatal Care, Health Education, Conocimientos, Actitudes y Prácticas en Salud, Prevención de Accidentes, Recién Nacido, Atención Prenatal, Educación en Salud

## Abstract

**Objective:**

To assess the knowledge, attitudes and practices of pregnant women regarding the prevention of risks and accidents involving their newborns.

**Methods:**

This was a cross-sectional study using a previously developed and validated instrument. The sample was non-probabilistic and intentional, defined based on a sample size calculation. The sociodemographic profile of the participants was analyzed using absolute and relative frequencies, followed by an assessment of the survey answers, which were considered satisfactory when they reached 70%. Subsequently, statistical analysis was performed using the chi-square test and Fisher’s exact test to assess association between the variables.

**Results:**

The study included 130 pregnant women, of whom 60.0% demonstrated low knowledge about risk of choking and 63.8% about risk of suffocation. Women in the third trimester, without a partner and who did not consume alcohol demonstrated greater knowledge of how to identify these risks. Of the pregnant women interviewed, 73.1% demonstrated adequate attitudes toward risk of suffocation, particularly among young women with a moderate income and having prenatal care with nurses. Regarding other factors, 94.6% demonstrated favorable attitudes. However, 67.7% of the pregnant women were not prepared for performing the Heimlich maneuver, indicating a lack of preparation for emergencies.

**Conclusion:**

Although the pregnant women demonstrated adequate about risks, their limited knowledge about choking and suffocation, combined with a lack of preparation for emergencies, highlights the need for educational interventions to improve safety and management of critical situations.

## Resumen

**Objetivo:**

Evaluar los conocimientos, actitudes y prácticas de mujeres embarazadas respecto a la prevención de riesgos y accidentes que afectan a sus recién nacidos.

**Métodos:**

Estudio cuantitativo transversal, con un instrumento previamente desarrollado y validado. La muestra fue no probabilística e intencional, definida mediante un cálculo del tamaño muestral. Se analizó el perfil sociodemográfico de las participantes mediante frecuencias absolutas y relativas, y posteriormente se realizó una evaluación de la encuesta, que se consideró satisfactoria al alcanzar el 70%. Posteriormente, se realizó un análisis estadístico mediante las pruebas de chi-cuadrado y exacta de Fisher para evaluar la asociación entre las variables.

**Resultados:**

El estudio incluyó a 130 mujeres embarazadas, de las cuales el 60,0% mostró un bajo nivel de conocimiento sobre el riesgo de atragantamiento y el 63,8% sobre el riesgo de asfixia. Las mujeres en el tercer trimestre, sin pareja y sin consumo de alcohol, mostraron un mayor conocimiento sobre cómo identificar estos riesgos. De las mujeres embarazadas entrevistadas, el 73,1% mostró actitudes adecuadas hacia el riesgo de asfixia, particularmente entre las mujeres jóvenes con ingresos moderados y acompañadas por enfermeras. En cuanto a otros factores, el 94,6% mostró actitudes favorables. Sin embargo, el 67,7% de las mujeres embarazadas no estaban preparadas para realizar la maniobra de Heimlich, lo que indica una falta de preparación para emergencias.

**Conclusión:**

Si bien las mujeres embarazadas demuestran actitudes adecuadas ante los riesgos, su limitado conocimiento sobre asfixia y sofocación, sumado a la falta de preparación para emergencias, resalta la necesidad de intervenciones educativas para mejorar la seguridad y la gestión en situaciones críticas.

**Ethical aspects**

This research respected ethical principles, having obtained the following approval data:

Research ethics committee: Centro Universitário Integral de Saúde Amaury de Medeiros

Opinion number: 6,466,738

Approval date: 27/10/2023

Certificate of submission for ethical appraisal: 58354222,6,0000,5191

Informed consent form: Obtained from all participants prior to collection.

## Introduction 

It has been found that pregnant women, especially first-time mothers, can turn to myths and cultural beliefs to promote care for their newborns (1). In this context, prenatal care plays a fundamental role in reducing risks and accidents, especially when provided by health professionals who promote an environment of active listening and health education, addressing both the gestational process and infant care. This approach can contribute to reducing infant mortality in Brazil (2).

Prenatal care enables the exchange of knowledge and the clarification of doubts, in addition to ensuring the mother’s understanding of the care that will be provided to her newborn, highlighting nurses as one of the main agents in promoting comprehensive and humanized care. Thus, educational actions during prenatal care can promote maternal and child health, in addition to preventing unfavorable outcomes for the newborn (3).

In this context, it is essential to clarify how to respond to risks that threaten the well-being of newborns, as many accidents are caused by carelessness on the part of parents or caregivers, such as suffocation, choking, falls and trauma (4). This is especially true when considering the difficulties that mothers may face and the myths surrounding interventions in situations of risk.

Foreign body aspiration, or choking, is one of the most serious accidents affecting newborns and can be classified as partial or total. When a baby is agitated, coughing, and crying, it may be due to partial choking. However, there are cases in which a baby is unable to cough or cry and has a purplish coloration, a sign of hypoxia, in addition to apnea. In these circumstances, this may be due to total choking, requiring the Heimlich maneuver to be performed as quickly as possible (5).

Another imminent risk for newborns is sudden infant death syndrome, which occurs mainly during sleep in babies under 1 year of age and is related to certain specific factors, namely: inadequate sleeping position (6), objects inside the crib, such as pillows, blankets and toys, and sharing beds and sofas (7,8). Still considering prevention of suffocation, the use of cords or necklaces around the baby’s neck should be avoided, especially until they are 4 months old (4).

Falls and trauma pose a constant danger, especially when young children come into contact with newborns. The Brazilian Society of Pediatrics (4) emphasizes the importance of prevention, recommending caution when allowing other children to have access to babies, as they may try to pick up newborns, imitating adult behavior.

Considering the risks inherent to newborns, the need to assess care provided is evident, since, due to the intrinsic fragility of this group, higher death rates occur in this age range. Undoubtedly, some deaths could be avoided when they are a consequence of the lack of guidance, during prenatal care, about newborn care, which could prevent actions that can put the newborn at risk (9).

This study took a comprehensive and integrated approach, as most existing literature focuses on specific categories of accidents and is mostly conducted with postpartum women. As it targets pregnant women, this study innovated by anticipating this preventive approach, increasing the time available for educational interventions and contributing to infant safety right from the prenatal stage.

This study sought to assess the knowledge, attitudes and practices of pregnant women regarding the prevention of risks and accidents to their newborn babies. 

## Methods 

### Design 

This was a cross-sectional study, linked to the research entitled *Health Indicators of Pregnant Women, Postpartum Women and Conceptuses in the Prenatal Care Service of a University Health Center*, which initially established the preparation and validation of a Knowledge, Attitudes and Practices Survey, following the steps proposed by Coluci, Alexandre and Milani (10). Based on this, this study presented the administration of the survey in practice.

### Setting 

The study was conducted at the outpatient unit of the Amaury de Medeiros Integrated University Health Center, located in the North Zone of Recife, within the territory of Health District II. The center is an Education and Health unit that is part of the University of Pernambuco Hospital Complex. Data were collected between November 2023 and January 2024.

### Participants 

The study included 130 pregnant women receiving prenatal care who were in their second or third trimester of pregnancy and agreed to participate. Those who were having their first appointment or who had a disability or mental disorder reported by a companion, making them unable to speak for themselves or their newborn, were not eligible for the study.

### Study size

The sample was non-probabilistic and intentional, establishing the sample size according to Barret & Kline (11), who reported that five respondents per survey item can be sufficient for a desired correlation. After the content and face validation process, 26 items needed to be kept, resulting in a sample of 130 pregnant women. 

Taking this number, a 10% expected loss was added for cases of dropout or failure to answer any of the survey items. In order to ensure greater statistical robustness, the sample was increased to 143 women.

### Variables 

The questionnaire was prepared in such a way that the variables to be analyzed formed data sets organized as follows:

Sociodemographic variables
Age group (<18 years; 18-35 years; >35 years);
Marital status (has a partner; does not have a partner);
Race/skin color (Brown (Brazilian mixed race); Black; White; Asian);
Family income (less than 1 minimum wage;
1 minimum wage; 2 or more minimum wages);
Schooling (levels 1, 2, 3 or 4).
Lifestyle habit variables
Alcohol consumer (yes; no);
Tobacco smoker (yes; no).
Health care variables
Prenatal care health professional (nurse; doctor).

In order to analyze the knowledge, attitudes and practices of the pregnant women, the following were considered to be satisfactory:

Knowledge
Know how to identify choking and perform the unchoking technique;
Know how to identify what can cause suffocation and how to prevent it;
Know the risks of sharing a bed with a newborn;
Know how to prevent falls and trauma and contact with pathogens.
Attitude
Believe that it is important to identify signs of choking and know how to perform the unchoking technique;
Believe that it is important to identify what can cause suffocation;
Believe that it is important not to put necklaces and cords around the newborn’s neck;
Believe that it is important not to use covers, blankets or objects in the newborn’s crib;
Believe that it is important not to put the newborn to sleep facedown;
Believe that it is important for the newborn not to share a bed;
Believe that it is important to always leave the newborn in the care of an adult;
Believe that it is important to prevent contact with pathogens.
Practice
Already having performed the unchoking maneuver or, if not, agree that they will be able to perform it;
Not putting necklaces and cords around the newborn’s neck;
Not using covers, blankets or objects in the newborn’s crib;
Putting the newborn to sleep tummy up;
Not sharing a bed with the newborn;
Not allowing a child to hold a newborn;
Only putting in the newborn’s bag the products and objects used by it.

### Data sources and measurement 

The data were collected by means of a questionnaire administered by two interviewers, using Google Forms, and are available at Scielo Data (12), together with the descriptive and statistical analyses.

Level of knowledge was measured based on the classification for assessing knowledge, attitudes and practices, adopting a cutoff point of 70% answer adequacy, according to criteria defined by similar studies (13,14). Thus, if a pregnant woman presented six or more satisfactory answers, the knowledge and practice domain would be considered adequate, and seven or more satisfactory answers would indicate an adequate attitude domain.

Answers ranged from “yes” to “no” in the knowledge domain. For attitudes, the 5-point Likert scale ranged from “not important” to “very important.” For practices, only one question ranged from “strongly disagree” to “strongly agree”. The remaining answers ranged from “never” to “very often”.

### Bias control

A potential selection bias was identified in that the study was conducted at a leading public hospital in Recife, which tends to attract pregnant women with specific characteristics. To minimize this bias, the study considered the participants’ sociodemographic profile and correlated it with their answers regarding knowledge, attitudes and practices, in order to achieve greater sample representation.

### Statistical analysis 

A simple descriptive analysis of the profile presented was performed. Therefore, the data were collected using a Microsoft Office Excel 2010 spreadsheet, where they were stored and assessed.

In order to investigate association between the pregnant women’s knowledge, attitudes and practices and sociodemographic, sexual, reproductive, lifestyle and health care variables, proportions were calculated using the Statistical Package for the Social Sciences version 20.0, and associations between variables were established using the chi-square test, which assumes the absence of cells with very low expected frequencies. In the absence of these assumptions, Fisher’s exact test was used, with a 5% significance level.

In cases where significant association was found, we calculated the value of Cramér’s V coefficient or the Phi coefficient, to determine the degree of association between the variables.

## Results 

Of the 130 pregnant women in the study, 104 (80.0%) were in the age range of 18-35 years, 21 (16.2%) were over 35 years old and 5 (3.8%) were minors. In addition, 67 women (52.3%) had a partner and 63 (47.7%) did not (Table 1).

**Table 1:** Distribution of the sociodemographic characteristics of pregnant women. Recife, 2024 (n=130)

Variables	n (%)
**Age group** (years)	
<18	5 (3.8)
18-35	104 (80.0)
>35	21 (16.2)
**Marital status**	
No partner	63 (47.7)
Partner	67 (52.3)
**Race/skin color**	
Brown	86 (66.1)
Black	29 (22.3)
White	8 (6.2)
Asian	7 (5.4)
**Family income (minimum wages^a^)**	
<1	32 (24.6)
1	70 (53.8)
≥2	28 (21.6)
**Schooling (levels)**	
1	15 (11.5)
2	25 (19.2)
3 or 4	90 (69.3)

^a^The minimum wage in 2024 was BRL 1,412.

Among the participants, 86 (66.1%) self-reported their race/skin color as Brown, 29 (22.3%) as Black, 8 (6.2%) as White and 7 (5.4%) as Asian. In addition, 70 pregnant women (53.8%) received 1 minimum wage, 32 (24.6%) received less than 1 minimum wage and 28 (21.6%) received 2 or more minimum wages. 

Schooling was classified according to the Institute of Applied Economic Research method (15). As such, 15 women (11.5%) had incomplete elementary education (level 1); 25 women (19.2%) had complete elementary education or had not finished high school (level 2); 79 women (60.8%) had completed high school or had not finished their degree (level 3); and 11 women (8.5%) had a degree (level 4).

The responses considered adequate and inadequate were highlighted, according to the reference value of 70% for satisfaction of knowledge, attitude and practice of pregnant women (Table 2). Aspects related to the identification and management of choking, in addition to the potential risks for cases of suffocation, presented a low level of knowledge (Table 2). 

**Table 2:** Satisfactory knowledge, attitudes and practices of pregnant women regarding risks to newborns, according to the survey items. Recife, 2024 (n=130)

Survey items	Knowledge			Attitude			Practice	
	Adequate	Inadequate		Adequate	Inadequate		Adequate	Inadequate
	n (%)	n (%)		n (%)	n (%)		n (%)	n (%)
Choking	52 (40.0)	78 (60.0)		123 (94.6)	7 (5.4)		42 (32.3)	88 (67.7)
Suffocation	47 (36.2)	83 (63.8)		95 (73.1)	35 (26.9)		88 (67.7)	42 (32.3)
Falls and trauma	99 (76.1)	31 (23.9)		123 (94.6)	7 (5.4)		78 (60.0)	52 (40.0)
Contact with pathogens	106 (81.5)	24 (18.5)		123 (94.6)	7 (5.4)		111 (85.4)	19 (14.6)

As for the attitude domain, the answers were mostly satisfactory, demonstrating that the participants agreed on how important it is to know how to save a newborn that is choking and to know how to identify signs of choking, in addition to the importance of knowing what could suffocate their babies.

However, when analyzing the practices of the pregnant women, low knowledge about identifying choking and the Heimlich maneuver resulted in inadequate answers, representing risks to the newborn babies’ lives.

In addition, the chi-square statistic was used to test the association between knowledge about newborn risks and accidents and sociodemographic, sexual, reproductive and lifestyle variables. Therefore, Table 3 details only the items that presented significant values ​​after the psychometric tests.

**Table 3:** Association between pregnant women’s knowledge about newborn risks and accidents and sociodemographic, sexual, reproductive and lifestyle habit variables, based on the Phi coefficient and Cramér’s V. Recife, 2024 (n=130)

Variables	Knowledge			
	Adequate	Inadequate	p-value	Association
	n (%)	n (%)		
**Do you know how to identify when your newborn is choking**?				
**Pregnancy trimester**			0.001	0.28^b^
Second	41 (31.5)	27 (20.8)		
Third	53 (40.8)	9 (6.9)		
**Do you know what to do to prevent your newborn from suffocating**?				
**Marital status**			0.034	0.19^b^
Partner	29 (22.3)	39 (30.0)		
No partner	38 (29.2)	24 (18.5)		
**Schooling** (levels)			0.005	0.28^c^
1	13 (10.0)	2 (1.5)		
2	15 (11.6)	10 (7.7)		
3 or 4	39 (30.0)	51 (39.2)		
**Do you know what can cause your newborn to suffocate**?				
**Alcohol consumer**			0.037^a^	-0.20^b^
Yes	0 (0.0)	4 (3.1)		
No	72 (55.4)	54 (41.5)		
**Do you know what to do to prevent your newborn from having falls and trauma**?				
**Marital status**			0.005	0.24^b^
Partner	45 (34.6)	23 (17.7)		
No partner	54 (41.5)	8 (6.2)		
**Pregnancy trimester**			0.017	0.21^b^
Second	46 (35.4)	22 (16.9)		
Third	53 (40.8)	9 (6.9)		

^a^Fisher’s exact test; ^b^Phi Coeffcient; ^c^Cramér’s V.

Regarding the item *Do you know how to identify when your newborn is choking*?, pregnant women in the second trimester had less adequate knowledge compared to those in the third trimester. 

As for the item *Do you know what to do to prevent your newborn from choking*?, Pregnant women with partners had more inadequate knowledge than those without a partner. Regarding schooling, pregnant women who met level 1 had greater adequate knowledge, while those with level education 3 or 4 had less adequate knowledge. Regarding the item *Do you know what can cause your newborn to choke*?, inadequate knowledge was greater among pregnant women who consumed alcohol compared to non-alcohol consumers.

As for the item *Do you know what to do to prevent your newborn from having falls and trauma*?, pregnant women with partners had greater inadequate knowledge, while pregnant women without a partner had greater adequate knowledge. Furthermore, regarding the gestational trimester, pregnant women in the third trimester had greater adequate knowledge when compared to those in the second trimester.

Attitudes regarding newborn risks and accidents were also associated with sociodemographic and health care variables (Table 4).

Living in other municipalities was associated with attitudes regarding the item *Do you think it’s important not to put covers, blankets or objects in your newborn’s crib*?. Pregnant women living in other municipalities demonstrated a greater degree of inadequate attitudes. Regarding the professional who conducted the prenatal care, there was a statistically significant association, which highlighted that pregnant women monitored by nurses exhibited more appropriate attitudes compared to those monitored by doctors.

In relation to the item *Do you think it’s important not to put your newborn to sleep facedown*?, pregnant women with family income of 2 or more minimum wages showed fewer adequate attitudes. As for the item *Do you think it’s important that your newborn should not sleep in the same bed as you*?, pregnant women under 18 years old had more inadequate attitudes.

We also sought to associate practices regarding newborn risks and accidents with sociodemographic, sexual, reproductive and health care variables (Table 5).

As for practices related to the item *Will you put necklaces or cords around your newborn’s neck*?, we found that pregnant women with family income corresponding to less than 1 minimum wage demonstrated lower adequate practices, while those with two or more minimum wages demonstrated greater adequate practices. Regarding gestational trimester, those in the second trimester achieved greater adequate practices, to the detriment of pregnant women in the third trimester, who were considered to have greater inadequate practices.

Regarding the item *Will you put covers, blankets or objects in your newborn’s crib*?, pregnant women who received prenatal care from nurses had greater adequate practices compared to those receiving care from doctors.

For the item *Will you put your newborn to sleep tummy up*?, pregnant women with partners had lower adequate practices, while those who reported not having a partner had higher adequate practices. Regarding the family income variable, pregnant women with income less than 1 minimum wage were found to have higher adequate practices, while pregnant women with two minimum wages or more, lower adequate practices were found.

As for the item *Will you let your newborn sleep in the same bed as you*?, pregnant women who lived in Recife showed greater adequate practices. Finally, regarding the item *Will you only keep the objects/products used by your newborn in your newborn’s bag*?, pregnant women in the second trimester showed greater adequate practices.

## Discussion 

Most of the pregnant women were found to demonstrate little knowledge about the risk of choking and suffocation, although they had adequate regarding the main risks to the newborn. However, regarding practices, especially in the case of choking, many were not prepared for performing the Heimlich maneuver, which reveals a significant gap in preparedness for emergencies, putting their babies’ safety at risk. It is noteworthy that, although the study, in the prenatal phase, provides time for reflection and preparation for future care, it does not allow for an assessment of how pregnant women would deal with these situations in practice, which limits the applicability of the results to the postpartum context.

Analysis of the survey results regarding pregnant women and risks to their newborns, revealed that the interviewees demonstrated little knowledge about the choking maneuver, which could hinder its practical application, if needed. In cases of total choking, agility in performing the technique is essential, since, if delayed, it can progress to cardiorespiratory arrest and result in the death of the newborn (16).

Regarding risk of suffocation, most demonstrated inadequate knowledge. Practices such as use of blankets and covers, improper sleeping positioning, bed-sharing, and the use of cords and necklaces around the baby’s neck are recognized risk factors for suffocation (4,7). Understanding these risks contributes to safer practices, avoiding adverse outcomes for the newborn.

With regard to the age of the study participants, the analysis revealed association, in that pregnant women under 18 years of age presented inadequatre attitudes towards bed-sharing. This practice, as emphasized, poses risks to the newborn’s life, especially with regard to sudden infant death syndrome. A literature review emphasizes that teenage pregnancy is among the risk factors for this syndrome (17), corroborating this study.

In relation to association with family income, we found that when asked about the position in which the newborn should sleep, there were inadequate attitudes and practices among pregnant women with better financial conditions. This is not in line with the literature, since, in a bibliographic analysis carried out, one of the main risk factors for sudden infant death was low socioeconomic status, due to the greater difficulty in implementing preventive measures, such as the appropriate sleeping position for the infant (18).

Regarding the use of necklaces and cords around newborns’ necks, which increase the risk of suffocation, pregnant women with better financial conditions presented better-than-expected answers. A descriptive study based on discussions with mothers about adherence to the practice of prone positioning for babies highlighted that our finding may be associated with greater access to information, since there is a relationship between higher family income and higher level of education (19).

With regard to questionnaire items on ways of preventing suffocation and sleeping posture, there was association with the presence or absence of a partner. For both these items, pregnant women with partners most often presented inadequate knowledge. However, compared to a quasi-experimental study conducted in a pediatric outpatient clinic at the Federal University of Ceará, there were more adequate answers among participants who had a partner (20). Thus, there is a clear need for couples to receive support and guidance during prenatal care.

Association was also found between schooling and knowledge about ways of preventing newborn suffocation. Thus, according to a theoretical-reflective study, mothers with low schooling are more vulnerable (21). However, our results disagreed with the literature, as our study showed that inadequate knowledge was greater among pregnant women with higher levels of schooling when compared to pregnant women with lower levels of schooling.

Also with regard to suffocation, questions were asked about what could cause suffocation in newborns. We found that pregnant women who consumed alcohol had inadequate knowledge. This is an important finding because, according to the study mentioned in the preceding paragraph, lifestyle habits can increase the chances of an unfortunate outcome in high-risk situations (21), such as mothers who report sleeping with their child after drinking alcohol.

In addition, we analyzed association between municipality and answers related to attitudes regarding items that should not be used in cribs and the practice of bed-sharing. We found that residents of the state capital (Recife) provided more adequate answers. This may be explained by a descriptive study conducted based on interviews with health professionals on the maternal practice of co-sleeping, which highlights that, in less populated regions, beliefs are more deeply rooted when compared to more populous cities, such as a state capital city (22).

We also found that, in relation to the item on recognizing signs of choking, pregnant women in the third trimester presented adequate answers when compared to those in the second trimester. This can be explained by a study of knowledge, attitudes and practices conducted with pregnant women from the West and Central-West regions of the state of Paraná, which highlighted that as the trimesters of pregnancy increased, the greater the knowledge presented (23).

However, regarding the practice of using necklaces and cords around newborns’ necks and sharing objects, the answers given by pregnant women in the third trimester were, in most cases, inadequate. This may have similarities with the findings of a study conducted with pregnant women undergoing prenatal care in a municipality in Paraná, in which the interviewees reported weakness in information provided during prenatal care (24), which was often superficial and restricted to the gestational phase.

A study of postpartum women whose prenatal care was provided exclusively at primary health care centers in a municipality in the interior region of the state of Minas Gerais highlights the importance of a multidisciplinary team in listening to pregnant women, seeking to understand their needs and, based on this, developing an adequate care plan (25). The fundamental role of nurses in prenatal care is highlighted, as, in this study, pregnant women monitored by this type of professional presented better answers for attitudes and practices regarding risk of suffocation. Nurses, as protagonists in health education, are essential in prenatal care, ensuring reliable information and guidance with humanized listening and holistic approach (26).

We found significant gaps in prenatal care for pregnant women, which should provide comprehensive, high-quality maternal and child health care. It is crucial to implement health education strategies, such as distributing pamphlets and holding lectures, to clarify pregnant women’s main concerns and encourage dialogue with healthcare professionals. This provides contributions to promoting knowledge, attitudes and practices among pregnant women with regard to preventing newborn risks and accidents.
