# Supplementary material for: Knowledge, attitudes and practices of pregnant women for preventing risks and accidents with their newborns: cross-sectional study, Recife, 2024
Source: Epidemiol Serv Saude. 2025 Dec 8;35:e20240822. doi: 10.1590/S2237-96222026v35e20240822.en (PMC12696818; doi:10.1590/S2237-96222026v35e20240822.en)
Supplement: Translation

# Translation: Conhecimento, atitude e prática da prevenção de riscos e de acidentes com o recém-nascido entre gestantes: estudo transversal, Recife, 2024

**DOI:** 10.1590/S2237-96222026v35e20240822.pt

**Authors:** Maria Eduarda da Silva Bastos, Ana Júlia Falcão Nascimento, Maria Tereza Ferreira Soares, Maria Benita Alves da Silva Spinelli, Sandra Trindade Low

**Affiliations:** 1 Universidade de Pernambuco, Recife, PE, Brasil

**Keywords:** Conhecimentos, Atitudes e Práticas em Saúde, Prevenção de Acidentes, Recém-nascido, Cuidado Pré-Natal, Educação em Saúde

## Abstract

**Objetivo:**

Avaliar os conhecimentos, as atitudes e as práticas das gestantes sobre a prevenção de riscos e de acidentes com o seu recém-nascido.

**Metodol:**

ogia: Tratou-se de estudo transversal, utilizando um instrumento previamente construído e validado. A amostra foi do tipo não probabilístico e intencional, definida a partir de cálculo amostral. Foi feita uma análise do perfil sociodemográfico das participantes, utilizando-se frequências absolutas e relativas, seguida da avaliação do inquérito, que foi considerado satisfatório ao atingir 70%. Posteriormente, realizou-se uma análise estatística, usando-se os testes qui-quadrado e exato de Fisher para verificar a associação entre as variáveis.

**Resultados:**

O estudo incluiu 130 gestantes, das quais 60,0% demonstraram baixo conhecimento sobre o risco de engasgo e 63,8% sobre o de sufocamento. Mulheres no terceiro trimestre, sem cônjuge e sem hábitos etílicos mostraram maior domínio sobre como identificar esses riscos. Das gestantes entrevistadas, 73,1% apresentaram atitudes adequadas sobre risco de sufocamento, destacando-se mulheres jovens, com renda média e acompanhadas por enfermeiros. Em relação aos outros itens, 94,6% apresentaram atitudes favoráveis. No entanto, 67,7% das gestantes não estavam preparadas para realizar a manobra de Heimlich, indicando falha na preparação para emergências.

**Conclusão:**

Embora as gestantes apresentem atitudes adequadas sobre riscos, o baixo conhecimento sobre engasgo e sufocamento, aliado à falta de preparo para emergências, destaca a necessidade de intervenções educativas para melhorar a segurança e o manejo em situações críticas.

**Aspectos éticos**

Esta pesquisa respeitou os princípios éticos, obtendo os seguintes dados de aprovação:

Comitê de ética em pesquisa: Centro Universitário Integral de Saúde Amaury de Medeiros

Número do parecer: 6.466.738

Data de aprovação: 27/10/2023

Certificado de apresentação de apreciação ética: 58354222.6.0000.5191

Registro do consentimento livre e esclarecido: Obtido de todos os participantes antes da coleta.

## Introdução 

É observado que as gestantes, principalmente primíparas, recorrem a mitos e a crenças culturais para promoção do cuidado ao seu recém-nascido (1). Nesse contexto, a assistência pré-natal desempenha um papel fundamental na redução de riscos e de acidentes, principalmente quando realizada por profissionais que promovem um ambiente de escuta ativa e educação em saúde, abordando tanto o processo gestacional quanto os cuidados com o bebê. Essa abordagem pode contribuir para a redução da mortalidade infantil no Brasil (2).

A assistência pré-natal torna possível a troca de saberes e o esclarecimento de dúvidas, além de assegurar a genitora quanto aos cuidados que serão dispensados ao recém-nascido, destacando o enfermeiro como um dos principais agentes na promoção de uma assistência integral e humanizada. Dessa forma, ações educativas no pré-natal são capazes de promover a saúde materno-infantil, além de prevenir desfechos desfavoráveis ao recém-nascido (3).

Nesse contexto, é imprescindível esclarecer como agir diante dos riscos que ameaçam a integridade do recém-nascido, pois muitos acidentes são causados por descuidos dos pais ou de cuidadores, como sufocamento, engasgo, quedas e traumas (4). Especialmente ao considerar as dificuldades que a genitora pode enfrentar e os mitos sobre intervenções em situações de risco.

A aspiração de corpo estranho, ou o engasgo, é um dos acidentes mais graves que podem acontecer com o recém-nascido e pode ser classificado como parcial ou total. Em ocasiões em que o bebê apresenta agitação, tosse e choro, implica-se o engasgo parcial. Porém, há casos em que ele é incapaz de tossir ou de chorar e apresenta coloração arroxeada, em sinal de hipóxia, além de apneia. Nessas circunstâncias, é representado o engasgo total, necessitando da realização da manobra de Heimlich o mais breve possível (5).

Outro risco iminente para neonatos é a síndrome da morte súbita do lactente, que ocorre principalmente durante o sono de bebês menores de 1 ano e está relacionada a alguns fatores específicos, a saber: posição inadequada ao dormir (6), objetos no interior do berço, como travesseiros, mantas e brinquedos, e compartilhamento de cama e de sofás (7,8). Ainda considerando a prevenção ao sufocamento, deve-se evitar o uso de cordões ou correntes no pescoço do bebê, especialmente até os 4 meses (4).

As quedas e os traumas representam um perigo constante, sobretudo quando crianças pequenas têm contato com o recém-nascido. A Sociedade Brasileira de Pediatria (4) destaca a importância da prevenção, recomendando cuidado com o acesso de outras crianças ao bebê, uma vez que elas podem tentar pegar o recém-nascido no colo, imitando comportamentos de adultos.

Considerando-se os riscos inerentes aos neonatos, evidencia-se a necessidade de avaliar os cuidados dispensados, pois, devido à fragilidade intrínseca desse grupo, maiores taxas de óbitos ocorrem nessa faixa etária. Assegura-se, também, que alguns óbitos poderiam ser evitados, quando são consequentes da falta de orientação, durante o pré-natal, acerca dos cuidados com o recém-nascido, o que poderia prevenir ações de risco (9).

Este estudo contou com uma abordagem abrangente e integrada, pois a maior parte da literatura existente foca categorias específicas de acidentes e é majoritariamente conduzida com puérperas. Direcionando-se às gestantes, este trabalho inovou ao antecipar esse olhar preventivo, ampliando o tempo disponível para intervenções educativas e contribuindo para a segurança do bebê desde o pré-natal.

Este estudou buscou avaliar os conhecimentos, as atitudes e as práticas das gestantes sobre a prevenção de riscos e de acidentes com o seu recém-nascido.

## Métodos 

### Delineamento do estudo

Tratou-se de estudo transversal, vinculado à pesquisa intitulada *Indicadores de Saúde das Gestantes, Puérperas e Conceptos no Pré-Natal de um Centro de Saúde Universitário*, a qual, inicialmente, estabeleceu a construção e a validação de um Inquérito de Conhecimento, Atitude e Prática, seguindo-se as etapas propostas por Coluci, Alexandre e Milani (10). A partir disso, este estudo apresentou a aplicação desse inquérito.

### Contexto 

A pesquisa foi conduzida na unidade ambulatorial do Centro Universitário Integrado de Saúde Amaury de Medeiros, localizado na zona Norte do Recife, no território do Distrito Sanitário de Saúde II. O centro é uma unidade de Educação e Saúde que integra o Complexo Hospitalar da Universidade de Pernambuco. Os dados foram coletados entre novembro de 2023 e janeiro de 2024.

### Participantes do estudo

O estudo contou com a participação de 130 gestantes, atendidas no pré-natal, que estavam no segundo ou no terceiro trimestre de gestação e que aceitaram participar da pesquisa. Não foram elegíveis para o estudo aquelas que estavam em primeira consulta ou que possuíam alguma incapacidade ou transtorno mental referido pelo acompanhante, tornando-as incapazes de responderem por si e pelo seu recém-nascido. 

### Tamanho do estudo

A amostra foi do tipo não probabilístico e intencional, estabelecendo-se o tamanho amostral de acordo com Barret e Kline (11), os quais relataram que cinco respondentes por item do inquérito pode ser o suficiente para uma correlação desejada. Após o processo de validação de conteúdo e aparente, houve a necessidade da permanência de 26 itens, resultando em uma amostra de 130 gestantes. 

A partir disso, acrescentaram-se 10% de perdas previstas para casos de desistência ou de ausência de resposta a algum dos itens do inquérito. Para garantir maior robustez estatística, conferiu-se a ampliação da amostra para 143 mulheres.

### Variáveis do estudo

Na elaboração do questionário, as variáveis a serem analisadas formaram conjuntos de dados organizados da seguinte maneira:

Sociodemográficas
Faixa etária (<18 anos; 18-35 anos; >35 anos);
Estado civil (com companheiro; sem companheiro);
Raça/cor da pele (parda; negra; branca; amarela);
Renda familiar (menos de 1 salário mínimo;
1 salário mínimo; 2 ou mais salários mínimos);
Escolaridade (níveis 1, 2, 3 ou 4).
Hábitos de vida
Etilista (sim; não);
Tabagista (sim; não).
Atendimento
Profissional que realiza o pré-natal (enfermeiro; médico).

Para analisar o conhecimento, a atitude e a prática das gestantes, considerou-se satisfatório:

Conhecimento
Saber identificar e realizar a técnica de desengasgo;
Saber identificar o que pode causar um sufocamento e como prevenir;
Saber os riscos da cama compartilhada;
Saber como prevenir quedas e traumas e contatos com patógenos.
Atitude
Considerar importante identificar sinais de engasgo e saber realizar a técnica de desengasgo;
Considerar importante identificar o que pode causar sufocamento;
Considerar importante não utilizar correntes e cordões no recém-nascido;
Considerar importante não utilizar mantas, cobertores e objetos no berço;
Considerar importante não colocar o recém-nascido para dormir de barriga para baixo;
Considerar importante não compartilhar a cama;
Considerar importante deixar o recém-nascido sempre com um adulto;
Considerar importante impedir o contato com patógenos.
Prática
Já ter realizado a manobra de desengasgo ou, caso não, concordar que conseguiria realizá-la;
Não utilizar correntes e cordões no recém-nascido;
Não utilizar mantas, cobertores e objetos no berço;
Colocar o recém-nascido para dormir de barriga para cima;
Não utilizar da cama compartilhada;
Não permitir que uma criança segure o recém-nascido;
Guardar na bolsa do recém-nascido apenas os produtos e os objetos utilizados por ele.

### Fontes de dados e mensuração

Os dados foram coletados mediante questionário aplicado por duas entrevistadoras, através do Google Forms, e estão disponíveis no Scielo Data (12), juntamente com as análises descritivas e estatísticas.

A mensuração do nível de conhecimento foi realizada com base na classificação para avaliar conhecimento, atitude e prática, adotando como ponto de corte a adequação de 70% nas respostas, conforme critérios definidos por estudos semelhantes (13,14). Assim, caso a gestante apresentasse seis ou mais respostas satisfatórias, o domínio de conhecimento e de prática seria considerado adequado, e sete ou mais respostas satisfatórias indicariam o domínio de atitude.

As respostas variaram entre “sim” e “não” no domínio de conhecimento. Para atitude, a escala Likert de 5 pontos ia de “não é importante” a “muito importante”. Já para prática, apenas uma questão seguia de “discordo totalmente” a “concordo totalmente”. As demais respostas variaram de “nunca” a “muito frequente”.

### Viés 

Identificou-se um viés potencial relacionado à seleção, isso porque o estudo foi realizado em um hospital público de referência em Recife, o qual tende a atrair gestantes com características específicas. Para minimizar esse viés, a pesquisa considerou o perfil sociodemográfico das participantes e o correlacionou a suas respostas sobre conhecimentos, atitudes e práticas, a fim de obter maior representatividade na amostra.

### Análise estatística

Foi realizada uma análise descritiva simples do perfil apresentado. Diante disso, realizou-se o levantamento dos dados utilizando-se a planilha eletrônica do Microsoft Office Excel 2010, onde eles foram armazenados e avaliados. 

A fim de investigar a associação do conhecimento, da atitude e da prática das gestantes com as variáveis sociodemográficas, sexuais, reprodutivas, de hábitos de vida e de atendimento, foram calculadas proporções através do programa Statistical Package for the Social Sciences versão 20.0 e realizadas associações entre as variáveis por meio do teste qui-quadrado, que pressupõe a inexistência de células com frequências esperadas muito baixas. Na ausência desses pressupostos, utilizou-se o teste exato de Fisher, com nível de significância de 5%.

Nos casos em que se constatou uma associação significativa, procedeu-se ao cálculo do valor do coeficiente V de Cramér ou do coeficiente Phi, utilizados para determinar o grau de associação entre as variáveis.

## Resultados 

Das 130 gestantes na pesquisa, 104 (80,0%) estavam na faixa etária de 18-35 anos, 21 (16,2%) tinham acima de 35 anos e 5 (3,8%) eram menores de idade. Além disso, 67 mulheres (52,3%) tinham companheiro e 63 (47,7%) não possuíam (Tabela 1).

**Tabela 1:** Distribuição das características sociodemográficas das gestantes. Recife, 2024 (n=130)

Variáveis	n (%)
**Faixa etária** (anos)	
<18	5 (3,8)
18-35	104 (80,0)
>35	21 (16,2)
**Estado civil**	
Sem cônjuge	63 (47,7)
Com cônjuge	67 (52,3)
**Raça/cor da pele**	
Parda	86 (66,1)
Negra	29 (22,3)
Branca	8 (6,2)
Amarela	7 (5,4)
**Renda familiar (salários mínimos^a^)**	
<1	32 (24,6)
1	70 (53,8)
≥2	28 (21,6)
**Escolaridade (níveis)**	
1	15 (11,5)
2	25 (19,2)
3 ou 4	90 (69,3)

^a^O valor do salário mínimo em 2024 foi R$ 1.412,00.

Dentre as participantes, 86 (66,1%) se autodeclararam pardas; 29 (22,3%), negras; 8 (6,2%), brancas; e 7 (5,4%), amarelas. Setenta gestantes (53,8%) recebiam 1 salário mínimo, 32 (24,6%) recebiam menos de 1 salário mínimo e 28 (21,6%) recebiam 2 ou mais salários mínimos. 

A escolaridade foi classificada conforme o Instituto de Pesquisa Econômica Aplicada (15). Assim, 15 mulheres (11,5%) possuíam ensino fundamental incompleto (nível 1); 25 mulheres (19,2%) finalizaram o ensino fundamental ou tinham ensino médio inacabado (nível 2); 79 mulheres (60,8%) concluíram o ensino médio ou não terminaram a graduação (nível 3); e 11 mulheres (8,5%) eram graduadas (nível 4).

Destacaram-se as respostas consideradas adequadas e inadequadas, segundo o valor de referência de 70% para satisfatoriedade de conhecimento, atitude e prática das gestantes (Tabela 2). Os aspectos relacionados à identificação e ao manejo do engasgo, além dos potenciais riscos para casos de sufocamento, apresentaram baixo nível de conhecimento (Tabela 2). 

**Tabela 2:** Satisfatoriedade de conhecimento, atitude e prática de gestantes sobre riscos ao recém-nascido, segundo os itens do inquérito. Recife, 2024 (n=130)

Itens do inquérito	Conhecimento			Atitude			Prática	
	Adequado	Inadequado		Adequado	Inadequado		Adequado	Inadequado
	n (%)	n (%)		n (%)	n (%)		n (%)	n (%)
Engasgo	52 (40,0)	78 (60,0)		123 (94,6)	7 (5,4)		42 (32,3)	88 (67,7)
Sufocamento	47 (36,2)	83 (63,8)		95 (73,1)	35 (26,9)		88 (67,7)	42 (32,3)
Quedas e traumas	99 (76,1)	31 (23,9)		123 (94,6)	7 (5,4)		78 (60,0)	52 (40,0)
Contato com patógenos	106 (81,5)	24 (18,5)		123 (94,6)	7 (5,4)		111 (85,4)	19 (14,6)

Já para o domínio da atitude, as respostas foram majoritariamente satisfatórias, demonstrando que as participantes concordavam sobre quão importante é saber desengasgar o recém-nascido e saber identificar se apresentam sinais de engasgo, além da importância de saber o que pode vir a sufocar o bebê.

No entanto, analisando-se as práticas das gestantes, observou-se que o baixo conhecimento sobre a identificação do engasgo e a manobra de Heimlich resultou em respostas inadequadas, representando riscos à vida do recém-nascido.

Ademais, a estatística qui-quadrado foi utilizada para testar a associação do conhecimento sobre riscos e acidentes ao recém-nascido com variáveis sociodemográficas, sexuais, reprodutivas e de hábitos de vida. Diante disso, detalhou-se, na Tabela 3, apenas itens que apresentaram valores de significância após a aplicação dos testes psicométricos.

**Tabela 3:** Associação entre conhecimento de gestantes sobre riscos e acidentes ao recém-nascido e variáveis sociodemográficas, sexuais, reprodutivas e hábitos de vida, com base no coeficiente Phi e V de Cramér. Recife, 2024 (n=130)

Variáveis	Conhecimento			
	Adequado	Inadequado	p-valor	Associação
	n (%)	n (%)		
**Sabe identificar quando o recém-nascido está engasgado?**				
**Trimestre da gestação**			0,001	0,28^b^
Segundo	41 (31,5)	27 (20,8)		
Terceiro	53 (40,8)	9 (6,9)		
**Sabe o que fazer para prevenir o sufocamento no recém-nascido?**				
**Estado civil**			0,034	0,19^b^
Com cônjuge	29 (22,3)	39 (30,0)		
Sem cônjuge	38 (29,2)	24 (18,5)		
**Escolaridade (níveis)**			0,005	0,28^c^
1	13 (10,0)	2 (1,5)		
2	15 (11,6)	10 (7,7)		
3 ou 4	39 (30,0)	51 (39,2)		
**Sabe identificar o que pode provocar o sufocamento do recém-nascido?**				
Etilista			0,037^a^	-0,20^b^
Sim	0 (0,0)	4 (3,1)		
Não	72 (55,4)	54 (41,5)		
**Sabe o que fazer para prevenir o risco de quedas e traumas ao recém-nascido?**				
**Estado civil**			0,005	0,24^b^
Com cônjuge	45 (34,6)	23 (17,7)		
Sem cônjuge	54 (41,5)	8 (6,2)		
**Trimestre da gestação**			0,017	0,21^b^
Segundo	46 (35,4)	22 (16,9)		
Terceiro	53 (40,8)	9 (6,9)		

^a^Teste exato de Fisher; ^b^Coeficiente Phi; ^c^V de Cramér.

Considerando-se o item *Sabe identificar quando o recém-nascido está engasgado*?, gestantes que estavam no 2º trimestre apresentaram menos conhecimento adequado, em comparação às do 3º trimestre. 

Para o item *Sabe o que fazer para prevenir o sufocamento no recém*-nascido?, gestantes com cônjuge apresentaram mais conhecimento inadequado do que as que não possuíam cônjuge. Quanto à escolaridade, gestantes que se adequaram ao nível 1 apresentaram maior conhecimento adequado, já as que se adequaram aos níveis 3 ou 4 apresentaram menor conhecimento adequado. Sobre o item *Sabe identificar o que pode provocar o sufocamento do recém*-nascido?, gestantes etilistas apresentaram maior conhecimento inadequado quando comparado às não etilistas.

Quanto ao item *Sabe o que fazer para prevenir o risco de quedas e traumas ao recém*-nascido?, as gestantes com cônjuge apresentaram maior conhecimento inadequado, enquanto as gestantes sem cônjuge apresentaram maior conhecimento adequado. Ademais, acerca do trimestre gestacional, as gestantes no 3º trimestre apresentaram maior conhecimento adequado quando comparado às que estavam no 2º trimestre.

Também se realizou associação da atitude sobre riscos e acidentes ao recém-nascido com as variáveis sociodemográficas e de atendimento (Tabela 4). 

**Tabela 4:** Associação entre atitude de gestantes sobre riscos e acidentes ao recém-nascido e variáveis sociodemográficas e de atendimento, com base no coeficiente Phi e V de Cramér. Recife, 2024 (n=130)

Variáveis	Atitude			
	Adequado	Inadequado	p-valor	Associação
	n (%)	n (%)		
**Considera importante não utilizar mantas, cobertores e objetos no berço do seu recém-nascido?**				
Município			0,034	0,23^c^
Recife	49 (37,7)	20 (15,4)		
Olinda	30 (23,1)	8 (6,1)		
Outros	11 (8,5)	12 (9,2)		
**Profissional que realiza o pré-natal**			0,028	-0,19^b^
Enfermeiro(a)	63 (48,4)	20 (15,4)		
Médico(a)	27 (20,8)	20 (15,4)		
**Considera importante não colocar o seu recém-nascido para dormir de barriga para baixo?**				
**Renda familiar (salários mínimos^a^)**			0,036	0,23^c^
<1	29 (22,3)	3 (2,4)		
1	57 (43,8)	13 (10,0)		
≥2	18 (13,8)	10 (7,7)		
**Você considera importante que o seu recém-nascido não durma na mesma cama com você?**				
**Faixa etária (anos)**			0,020	0,25^c^
<18	1 (0,7)	4 (3,1)		
18-35	77 (59,2)	27 (20,8)		
>35	17 (13,1)	4 (3,1)		

^a^O valor do salário mínimo em 2024 foi R$ 1.412,00; ^b^Coeficiente Phi; ^c^V de Cramér.

O fato de morar em outros municípios foi associado à atitude no item *Considera importante não utilizar mantas, cobertores e objetos no berço do seu recém*-nascido?. A quantidade de gestantes que moravam em outros municípios demonstrou maior atitude inadequada. Quanto ao profisisonal que realizou o pré-natal, houve associação estatisticamente significativa, a qual enfatizou que gestantes acompanhadas por enfermeiros apresentaram mais atitudes adequadas, em comparação àquelas acompanhadas por médicos.

Em relação ao item *Considera importante não colocar o seu recém-nascido para dormir de barriga para baixo*?, gestantes com renda familiar de 2 ou mais salários apresentaram menos atitudes adequadas. No item *Você considera importante que o seu recém-nascido não durma na mesma cama com você*?, gestantes menores de 18 anos apresentaram mais atitudes inadequadas.

Buscou-se também associar a prática sobre riscos e acidentes ao recém-nascido com as variáveis sociodemográficas, sexuais, reprodutivas e de atendimento (Tabela 5).

**Tabela 5:** Associação entre prática de gestantes sobre riscos e acidentes ao recém-nascido e variáveis sociodemográficas, sexuais, reprodutivas e de atendimento, com base no coeficiente Phi e V de Cramér. Recife, 2024 (n=130)

Variáveis	Prática			
	Adequado	Inadequado	p-valor	Associação
	n (%)	n (%)		
**Você utilizará correntes ou cordões no pescoço do seu recém-nascido?**				
**Renda familiar (salários mínimos^a^)**			0,026	0,24^c^
<1	17 (13,1)	15 (11,5)		
1	47 (36,1)	23 (17,7)		
≥2	24 (18,5)	4 (3,1)		
**Trimestre da gestação**			0,025	-0,20^b^
Segundo	52 (40,0)	16 (12,3)		
Terceiro	36 (27,7)	26 (20,0)		
**Você utilizará mantas, cobertores e objetos no berço do seu recém-nascido?**				
**Profissional que realiza o pré-natal**			0,004	-0,25^b^
Enfermeiro(a)	39 (30,0)	44 (33,8)		
Médico(a)	10 (7,7)	37 (28,5)		
**Você colocará o seu recém-nascido para dormir de barriga para cima?**				
**Estado civil**			0,014	0,22^b^
Com cônjuge	27 (20,8)	41 (31,5)		
Sem cônjuge	38 (29,2)	24 (18,5)		
**Renda familiar (salários mínimos^a^)**			0,016	0,25^c^
<1	21 (16,1)	11 (8,5)		
1	36 (27,7)	34 (26,2)		
≥2	8 (6,1)	20 (15,4)		
**Você deixará o seu recém-nascido dormir na mesma cama com você?**				
Município			0,026	0,24^c^
Recife	30 (23,1)	39 (30,0)		
Olinda	12 (9,2)	26 (20,0)		
Outros	3 (2,3)	20 (15,4)		
**Você guardará na bolsa do recém-nascido apenas os objetos/produtos utilizados por ele?**				
**Trimestre da gestação**			0,050	-0,17^b^
Segundo	62 (47,7)	6 (4,6)		
Terceiro	49 (37,7)	13 (10,0)		

^a^O valor do salário mínimo em 2024 foi R$ 1.412,00; ^b^Coeficiente Phi; ^c^V de Cramér.

Para a prática relacionada ao item *Você utilizará correntes ou cordões no pescoço do seu recém*-nascido?, observou-se que as gestantes com renda familiar correspondente a menos de 1 salário mínimo demonstravam menor prática adequada, enquanto as com 2 salários mínimos ou mais apresentavam uma maior prática adequada. Em relação ao trimestre gestacional, as englobadas no 2º trimestre alcançaram maior prática adequada, em detrimento das gestantes do 3º trimestre, às quais se considerou uma maior prática inadequada.

No que se diz respeito ao item *Você utilizará mantas, cobertores e objetos no berço do seu recém*-nascido?, as gestantes que foram atendidas no pré-natal por enfermeiros obtiveram maior prática adequada em comparação às atendidas por médicos.

Para o item *Você colocará o seu recém-nascido para dormir de barriga para cima*?, gestantes com cônjuge apresentaram menor prática adequada, enquanto as que relataram não ter um cônjuge obtiveram maior prática adequada. Quanto à variável de renda familiar, para as gestantes com renda menor que 1 salário mínimo, foi observada maior prática adequada. Para as gestantes com 2 salários mínimos ou mais, foi observada menor prática adequada.

No item *Você deixará o seu recém-nascido dormir na mesma cama com você*?, as gestantes que residiam em Recife demonstraram maior prática adequada. Por fim, para o item *Você guardará na bolsa do recém-nascido apenas os objetos/produtos utilizados por ele*?, gestantes no 2º trimestre apresentaram maior prática adequada.

## Discussão 

Evidenciou-se que a maioria das gestantes demonstrou baixo conhecimento sobre o risco de engasgo e de sufocamento, embora tivessem atitudes adequadas em relação aos principais riscos para o recém-nascido. Contudo, em relação à prática, sobretudo no caso de engasgo, muitas não estavam preparadas para realizar a manobra de Heimlich, o que revela uma lacuna importante na preparação para emergências, colocando em risco a segurança do bebê. Nota-se que, embora o estudo, na fase pré-natal, proporcione o tempo para reflexão e preparação para os cuidados futuros, ele não permite avaliar como as gestantes lidariam com essas situações na prática, o que limita a aplicabilidade dos resultados ao contexto pós-parto.

Seguindo a análise do inquérito apresentado pelas gestantes sobre os riscos ao recém-nascido, observou-se que as entrevistadas demonstraram baixo conhecimento sobre a manobra de desengasgo, o que poderia desfavorecer sua aplicação prática, caso necessário. Em caso de engasgo total, a agilidade na técnica é indispensável, já que, em casos de demora, ele pode evoluir para uma parada cardiorrespiratória e acarretar óbito do recém-nascido (16).

Quanto ao risco para sufocamento, a maior parte demonstrou um conhecimento inadequado. Práticas como o uso de mantas e de cobertores, o posicionamento inadequado para dormir, o compartilhamento da cama e o uso de cordões e de colares no pescoço do bebê são fatores de risco reconhecidos para o sufocamento (4,7). Entende-se que compreender esses riscos contribui para uma prática mais segura, evitando desfechos desfavoráveis ao recém-nascido.

Em relação à idade das participantes do estudo, durante a associação realizada, percebeu-se que gestantes menores de 18 anos apresentaram atitudes inadequadas quanto à prática da cama compartilhada. Essa prática, como ressaltado, traz riscos à vida do recém-nascido, sobretudo quanto à síndrome da morte súbita do lactente. Um levantamento bibliográfico realizado enfatiza que, dentre os fatores de risco para essa síndrome, está a gravidez na adolescência (17), corroborando este estudo.

Percebeu-se que, quanto à associação com a renda familiar, ao ser questionado sobre a posição que o recém-nascido deve dormir, houve atitude e prática inadequadas das gestantes com melhores condições financeiras. Isso vai de encontro à literatura, pois, em análise bibliográfica realizada, um dos principais fatores de risco para morte súbita do lactente foi o baixo nível socioeconômico, devido à maior dificuldade de aplicar medidas preventivas, como a posição adequada para o lactente dormir (18).

Já em relação ao uso de correntes e de cordões no recém-nascido, os quais potencializam os riscos para sufocamento, gestantes com melhores condições financeiras apresentaram respostas adequadas superiores ao esperado. Um estudo descritivo realizado a partir de debates com genitoras sobre a adesão à prática da posição prona dos bebês ressaltou que esse achado pode estar associado ao maior acesso a informações, uma vez que há uma relação entre maior renda familiar e maior nível de escolaridade (19).

Quanto aos itens sobre formas para prevenir o sufocamento e a posição postural para dormir, houve associação com a presença ou não do cônjuge. Para ambos, gestantes com cônjuge apresentaram, na maioria das vezes, conhecimento inadequado. No entanto, em comparação a um estudo quase-experimental, realizado em um ambulatório, especializado em pediatria, da Universidade Federal do Ceará, houve respostas mais adequadas entre participantes que possuíam cônjuge (20). Assim, percebe-se a necessidade de que esses casais recebam suporte e orientações durante o pré-natal.

Observou-se também associação entre escolaridade e conhecimento acerca de formas de prevenir o sufocamento no recém-nascido. Assim, segundo um estudo teórico-reflexivo, mães com baixa escolaridade apresentam maior vulnerabilidade (21). No entanto, os resultados apresentaram discordância com a literatura, pois se evidenciou, neste estudo, que gestantes com maiores níveis de escolaridade apresentaram mais conhecimento inadequado quando comparado a gestantes com menor nível de escolaridade.

Seguindo essa perspectiva, questionou-se sobre o que poderia vir a causar um sufocamento no recém-nascido. Diante disso, percebeu-se que gestantes etilistas apresentaram conhecimento inadequado. Esse é um achado importante, pois, ainda de acordo com o estudo, os hábitos de vida podem aumentar as chances de um desfecho infeliz diante de situações de alto risco (21), como o caso de mães que relatam dormir com o filho após a ingestão de bebidas alcoólicas.

Ademais, analisou-se a associação do município com as respostas relacionadas à atitude quanto aos itens que não devem ser utilizados no berço e à prática da cama compartilhada. Percebeu-se que moradoras da capital apresentaram mais respostas adequadas. Isso pode ser justificado por estudo descritivo realizado a partir de entrevistas com profissionais da área da saúde sobre a prática do coleito pelas genitoras, o qual destaca que, em regiões menos populosas, as crenças são mais enraizadas quando comparadas às cidades mais populosas, como uma capital (22). 

Observou-se também que, em relação ao item sobre reconhecer sinais de engasgo, gestantes no 3º trimestre apresentaram respostas adequadas quando comparadas às que estavam no 2º trimestre. Isso pode ser justificado a partir de um estudo de conhecimento, atitude e prática realizado com gestantes da região Oeste e Centro-Oeste do Paraná, o qual salientou que, quanto maior o trimestre de gestação, maior é o conhecimento apresentado (23).

No entanto, quanto à prática que inclui o uso de correntes e de cordões no recém-nascido e o compartilhamento de objetos, as respostas de gestantes no 3º trimestre foram, na maioria das vezes, inadequadas. Isso pode estar relacionado com os achados de um estudo realizado com gestantes em acompanhamento pré-natal de um município do Paraná, em que as entrevistadas relataram fragilidade nas informações repassadas durante o pré-natal (24), sendo essas, muitas vezes, superficiais e restritas à fase gestacional.

Um estudo, realizado com puérperas cujo pré-natal foi feito exclusivamente em unidades básicas de saúde em um município do interior de Minas Gerais, destaca a importância da equipe multidisciplinar na escuta da gestante, buscando entender suas necessidades e, a partir disso, elaborar um plano de cuidados adequado (25). Destaca-se o papel fundamental do enfermeiro no pré-natal, visto que, neste estudo, as gestantes acompanhadas por esse tipo de profissional apresentaram melhores respostas em atitude e prática sobre o risco de sufocamento. O enfermeiro, como protagonista na educação em saúde, é essencial na assistência pré-natal, garantindo informações seguras e orientações com escuta humanizada e olhar holístico (26).

Notam-se as lacunas existentes na assistência às gestantes atendidas no pré-natal, o qual deveria dispor de integralidade e qualidade para a saúde materno-infantil. É crucial implementar estratégias de educação em saúde, como distribuição de panfletos e palestras, para esclarecer as principais dúvidas das gestantes e incentivar o diálogo com os profissionais de saúde. Fornecem-se, dessa maneira, contributos para promoção de conhecimento, atitude e prática de gestantes na prevenção de riscos e de acidentes ao recém-nascido.
